# Tetrasodium EDTA for the prevention of urinary catheter infections and blockages†

**DOI:** 10.1039/d2ra06418a

**Published:** 2023-01-11

**Authors:** Jessica V. Moore, Doyoung Kim, Nicola J. Irwin, Jeffrey D. Rimer, Colin P. McCoy

**Affiliations:** a School of Pharmacy, Queen's University Belfast 97 Lisburn Road Belfast BT9 7BL Northern Ireland UK c.mccoy@qub.ac.uk; b Department of Chemical and Biomolecular Engineering, University of Houston Houston TX 77204 USA

## Abstract

Long-term catheterised individuals are at significant risk of developing catheter-associated urinary tract infections (CAUTIs), with up to 50% of patients experiencing recurrent episodes of catheter encrustation and blockage. Catheter blockage is a result of accumulation of carbonate apatite and struvite formed upon precipitation of ions within urine due to an infection-induced rise in pH. The aim of this study was to investigate the antimicrobial and anti-encrustation activities of tetrasodium ethylenediaminetetraacetic acid (tEDTA) to evaluate its potential efficacy in preventing CAUTIs and catheter blockages. The antimicrobial activity of tEDTA against uropathogens was assessed using time kill assays performed in artificial urine (AU). Crystallisation studies and *in vitro* bladder model assays were conducted to investigate the effect of tEDTA on struvite crystallisation and catheter blockage. tEDTA displayed bacteriostatic activity against *Proteus mirabilis* and prevented precipitation of ions in the AU. Crystallisation studies confirmed tEDTA inhibits struvite nucleation and growth *via* Mg^2+^ chelation with 7.63 mM tEDTA, equimolar to the concentration of divalent cations in AU, preventing the formation of crystalline deposits and blockage of Foley catheters for ≥168 h. The promising chelating abilities of low tEDTA concentrations could be exploited to inhibit encrustation and blockage of indwelling catheters. The fundamental research presented will inform our future development of an effective tEDTA-eluting catheter coating aimed at preventing catheter encrustation.

## Abstract 2

Tetrasodium EDTA is an effective chelating agent that prevents struvite crystallisation in *P. mirabilis*-infected artificial urine, allowing catheters exposed to low tEDTA concentrations to remain unblocked after 168 h.

## Graphical abstract

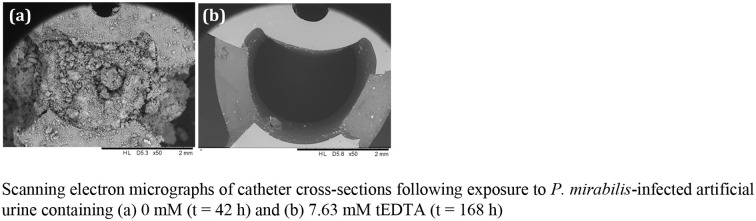

## 1. Introduction

Urinary catheters, either for intermittent or long-term use, are one of the most frequently employed medical devices in clinical care.^1,2^ Bacterial colonisation of catheters, however, can lead to the development of catheter-associated urinary tract infections (CAUTIs), which represent the most common healthcare-associated infection and account for up to 80% of all nosocomial UTIs.^1,3,4^ CAUTIs not only increase patient morbidity but, if left untreated, can lead to serious, life-threatening complications such as pyelonephritis and urosepsis.^5^

Understanding the pathogenesis of CAUTIs and the cascade of events that lead to catheter blockage are important to tackle this global healthcare issue. Despite the use of aseptic measures during catheter insertion, microbial contamination may be considered inevitable, with the introduction of pathogens into the typically sterile urinary tract.^5^ Of the range of microbes that colonise the catheter surface, urease-producing bacteria such as *Proteus mirabilis* are particularly problematic due to their role in the formation of crystalline biofilms and subsequent encrustation of the catheter. Urease-induced hydrolysis of urea in the urine to ammonia and carbon dioxide causes elevation of urinary pH, with resultant precipitation of polyvalent ions.^5,6^ This can lead to the production of an amorphous carbonate apatite precipitate and the nucleation of magnesium ammonium phosphate hexahydrate (struvite) crystals, which can agglomerate together to form infectious urinary stones.^6,7^ Struvite crystals can grow into large staghorn calculi which cause significant trauma to the bladder and/or urethra and disrupt the flow of urine.^7,8^

**Fig. 1:**
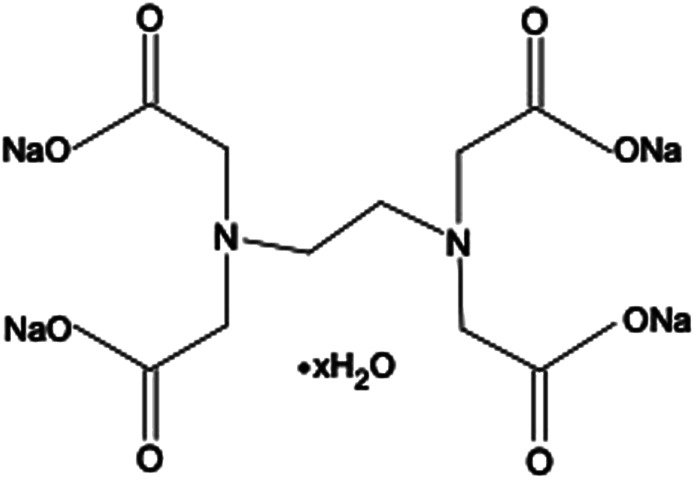
Chemical structure of ethylenediaminetetraacetic acid tetrasodium salt hydrate (tEDTA).

Ethylenediaminetetraacetic acid (EDTA) and its salt forms are well-established hexadentate chelating agents which have been used in a range of clinical and non-clinical applications, including treatment of heavy metal poisoning, and preservation and stabilisation purposes in food, cosmetic, pharmaceutical, and ophthalmic products.^9,10^ In addition, the chelating abilities of EDTA impart this agent with antimicrobial and antibiofilm properties. EDTA chelates cations present in bacterial cell walls and in biofilm matrices, leading to their destabilisation. A further mechanism by which EDTA can adversely affect bacterial viability is through chelation of cations essential for bacterial survival and growth.^9,11–13^ EDTA has been investigated both as an individual antimicrobial/antibiofilm agent and as an agent that can potentiate the activity of other antimicrobials.^9–11^ The tetrasodium salt form of EDTA (tEDTA), as shown in Fig. 1, has been reported to exhibit effective antimicrobial and antibiofilm activities.^9,14^ Furthermore, EDTA demonstrates promising capacity for the prevention of struvite crystallisation and urinary catheter encrustation through the chelation of Ca^2+^ and Mg^2+^ ions in the urine and resultant reduction in crystal formation owing to decreased supersaturation.^7,15,16^

Herein we investigate the antimicrobial and anti-encrustation activities of tEDTA against uropathogens to evaluate the dual efficacy of this agent for combatting CAUTIs and associated catheter blockages. The antimicrobial activity of tEDTA against uropathogens was assessed using time kill assays performed in artificial urine (AU). The effect of tEDTA on struvite nucleation and growth, and subsequent catheter encrustation and blockage, was examined in crystallisation studies and *in vitro* bladder model assays, respectively.

## 2. Materials and methods

Ethylenediaminetetraacetic acid tetrasodium salt hydrate (≥99.0%) (tEDTA), calcium chloride dihydrate, magnesium chloride hexahydrate (≥99.0%), sodium chloride (≥99.0%), sodium sulfate (≥99.0%), trisodium citrate dihydrate (≥99.0%), potassium chloride (≥99.0%), ammonium chloride (≥99.5%) and urea were obtained from Sigma-Aldrich (Dorset, UK). Creatinine (98%), sodium oxalate (≥99.5%) and potassium dihydrogen phosphate (≥98.0%) were purchased from Alfa Aesar (Heysham, UK). Quarter-strength Ringer's solution (QSRS), phosphate-buffered saline (PBS), tryptone soya broth (TSB), Mueller-Hinton broth (MHB) and agar were purchased from Oxoid Ltd (Hampshire, UK). BARD silicone, 14-channel male catheters and URIPLAN 2 L drainable bed bags (98 cm inlet) were purchased from BARD Ltd (Crawley, UK). *Proteus mirabilis* ATCC 51286 and *Staphylococcus aureus* ATCC 29213 were purchased from LGC Standards (Middlesex, UK), and *Escherichia coli* NSM59 was obtained from Dr Jones (University of Bath, UK). Deionised water used in bulk crystallisation assays was purified with an Aqua Solutions RODI-C-12A purification system (18.2 MΩ).

### 2.1. Determination of tEDTA kill kinetics

Time kill assays were performed in AU to assess the kinetics of tEDTA activity against *S. aureus*, *P. mirabilis*, and *E. coli* in biologically-relevant media. The AU composition (Table 1) was based on the recipe described by Griffith *et al.* and the time kill method was performed according to Clinical and Laboratory Standards Institute (CLSI) guidelines.^17–19^ Two tEDTA concentrations of 7.63 and 28 mM were tested. The required mass of tEDTA was dissolved in 49.75 mL AU immediately before starting the experiment to minimise premature chelation of Ca^2+^ and Mg^2+^ in the AU, and the solution was filter sterilised using a 0.45 μm syringe filter. Control flasks were similarly prepared without tEDTA. The bacterial inoculum in the logarithmic growth phase was adjusted with AU to an optical density at 550 nm (OD_550nm_) equivalent to 1 × 10^8^ CFU mL^−1^, as verified by viable count. Aliquots (0.25 mL) were added to the flasks to give an inoculum density of *ca.* 5 × 10^5^ CFU mL^−1^. Flasks were incubated at 37 °C at 100 rpm and samples removed after 0, 1, 2, 3, 4, 6 and 24 h of exposure. Ten-fold serial dilutions were performed in a neutraliser solution containing 3.20 mM Ca^2+^; 2.66 mM calcium chloride dihydrate dissolved in QSRS (containing 0.54 mM Ca^2+^).^19–21^ Diluted samples (20 μL) were plated onto nutrient agar (NA), or low swarm agar (LSWA) for *P. mirabilis*, and viable cells enumerated following 24 h incubation at 37 °C using the Miles and Misra method.^22^ The pH of the media was recorded after 0, 2, 4, 6 and 24 h incubation with a Hanna HI 5221 pH meter and micro HI1083B electrode (Hanna Instruments Ltd UK). Assays were repeated on three independent occasions. Bacteriostatic and bactericidal activities were respectively defined as a <3 log_10_ reduction and a ≥ 3 log_10_ reduction in bacterial density after 24 h exposure to tEDTA, relative to the starting inoculum.^18,19^

**Table 1:** AU composition based on the recipe reported by Griffith *et al.*^17^^a^

Chemical	Concentration (g L^−1^)
Calcium chloride dihydrate	0.65
Magnesium chloride hexahydrate	0.65
Sodium chloride	4.60
Sodium sulfate	2.30
Trisodium citrate dihydrate	0.65
Sodium oxalate	0.02
Potassium dihydrogen phosphate	2.80
Potassium chloride	1.60
Ammonium chloride	1.00
Urea	25.00
Creatinine	1.10
Tryptone soya broth	0.01

a The pH of the AU was adjusted to pH 5.7–5.8 using 1 M sodium hydroxide solution and the AU was filter sterilised using a 0.45 μm filter.

### 2.2. Bulk struvite crystallisation studies

#### 2.2.1. Analysis of crystal formation and morphology

The bulk crystallisation protocol followed the procedures reported in previous work by Kim *et al.*^8^ Briefly, the effects of tEDTA were investigated in growth solutions (10 mL) with a composition of 7 mM MgCl_2_·6H_2_O : 7 mM NH_4_H_2_PO_4_ : 150 mM NaCl : X mM tEDTA (where X = 0, 1, 3, 5 and 7) prepared at pH 8.60 (±0.03) using 1 M NaOH. Solutions were stirred for 15 min at 1200 rpm and left under static conditions at ambient temperature (20 °C) for 24 h before analysis by optical microscopy using a Leica DMi8 instrument. The crystals formed were retrieved from solution using a Büchner filtration method. Scanning electron microscopy (SEM) using a FEI 235 dual-beam focused ion beam instrument was employed to analyse the dried crystals. SEM samples were prepared by placing the crystals onto carbon tape and coating with 15–20 nm gold to reduce electron beam charging. Additionally, the dried crystals were analysed with a Siemens D5000 X-ray powder diffractometer (XRD) using a CuKα source (40 kV, 30 mA). Struvite formation was confirmed using XRD reference patterns provided by the database of the RRUFF Project with ID:R050540.1. Crystals from a minimum of six growth solutions were analysed.

#### 2.2.2. Analysis of crystallisation kinetics

Growth solution pH was recorded over time to assess the effect of tEDTA concentration on the rate of struvite crystallisation. The 10 mL growth solution with initial pH of 8.60 (±0.03) was prepared as described in section 2.2.1. and stirred continuously. The pH was automatically recorded at 0.5 min intervals up to 600 min by an Orion 3-Star Plus pH benchtop meter equipped with a ROSS Ultra electrode (8102BNUWP). The pH change of the growth solution was used as an indicator of struvite crystallisation (including the effects of both nucleation and crystal growth). The extent of reaction (EOR) was assessed according to the following equation:1
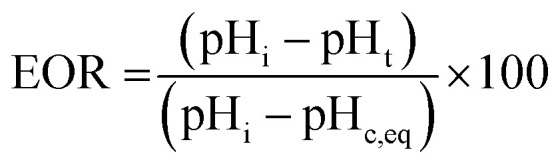
where pH_*i*_ is the initial pH (8.60), pH_*t*_ is the pH at time *t*, and pH_*c,eq*_ is the final equilibrium pH (pH 7.42) achieved by struvite formation free of additive (control).

### 2.3. Struvite microfluidics growth analysis

A microfluidics device was used to examine struvite growth following the set-up and protocol reported in previous work.^8,23^ Growth solutions for microfluidics studies were prepared with molar concentrations of 2.5 mM MgCl_2_·6H_2_O : 2.5 mM NH_4_H_2_PO_4_ : X mM tEDTA at pH 8.60. The growth solution was delivered to the microchannel at flow rates of 24 mL h^−1^ using a dual syringe pump (Chemyx, Fusion 4000) and two syringes (plastic BD syringe, 30 mL) with an in-line mixing configuration. Solution 1 contained MgCl_2_·6H_2_O mixed with NH_4_H_2_PO_4_, and solution 2 contained NaOH and tEDTA. For *in situ* time-resolved studies, images were acquired every 5 min at multiple positions along the microfluidics channel. The growth rate was measured by linear regression of crystal length along the *a⃑*, *b⃑* and *c⃑* directions *versus* time data. The effects of various concentrations of tEDTA were quantitatively assessed through calculation of the reduced growth rate (RGR) defined as:2
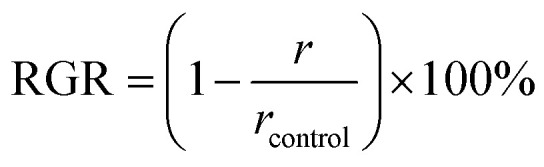
where *r* and *r*_control_ are growth rates in the presence and absence of tEDTA, respectively.

### 2.4. *In vitro* bladder model assays

**Fig. 2:**
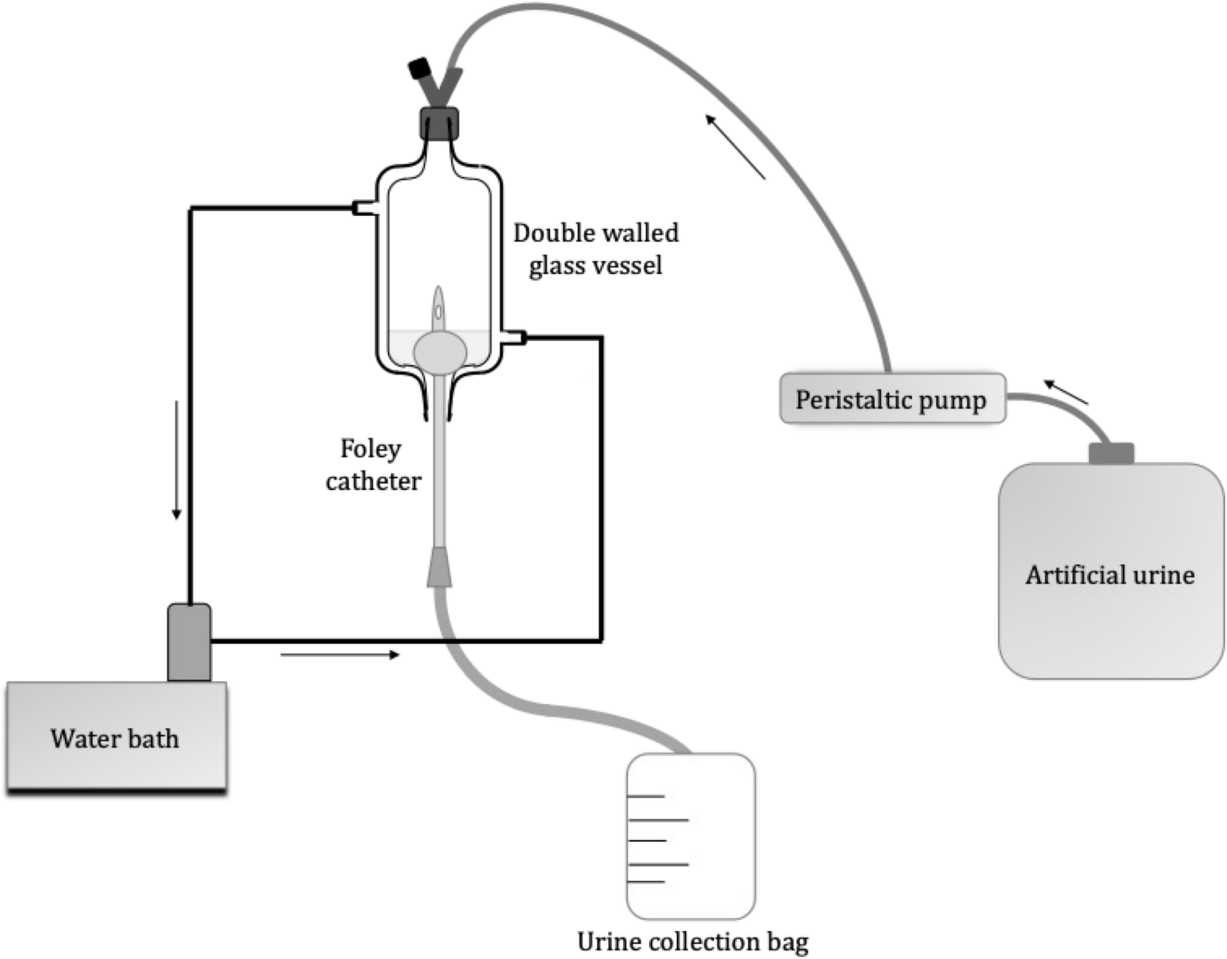
Schematic diagram of the *in vitro* bladder model assembly. A silicone 14 channel male Foley catheter was inserted into the bladder and held in place by inflating the balloon. The water bath supplied water at a temperature of 37 °C to the water jacket surrounding the inner chamber of the glass vessel. The peristaltic pump supplied sterile AU, stored in a 10 L sterile carboy, to the bladder at a rate of 0.75 mL min^−1^. When the level of AU in the bladder reached the level of the catheter eyehole, the urine drained from the bladder and was collected in the attached urine collection bag.

The assembly of the *in vitro* bladder model (Fig. 2), was similar to that described by Nzakizwanayo *et al.*^24^. The composition of AU prepared is shown in Table 1; however, the TSB concentration increased from 0.001 to 0.1% w/v to maintain viability of the high *P. mirabilis* inoculum within the flow model. Filter-sterilised solutions of 7.63 and 28 mM tEDTA were added to AU immediately before starting the experiment to minimise premature chelation of ions. Control urine was prepared without tEDTA. *P. mirabilis* in the logarithmic growth phase was centrifuged at 3000 rpm for 12 min, the supernatant discarded, and the bacterial pellet resuspended in AU. The suspension was adjusted to an OD_550nm_ equivalent to *ca.* 1 × 10^9^ CFU mL^−1^, as verified by viable count. This inoculum (10 mL) was aseptically added to each bladder 1 h before starting the flow of urine (*t* = 0 h) to allow the inoculum to establish within the bladder models. The urine flow to each bladder was maintained at a rate of 0.75 mL min^−1^ for 168 h or until catheter blockage. Samples (1 mL) were taken from the bladders at *t* = 0 h, daily, following catheter blockage, and at *t* = 168 h if blockage had not occurred, for measurement of pH and determination of bacterial viability. The total volume of urine collected in the drainage bags was used to calculate the time of blockage for each catheter,3
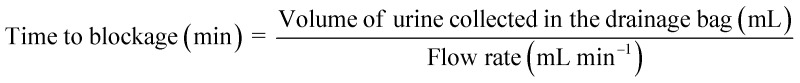


At the time of catheter blockage, or at 168 h if not blocked, the catheters were removed from the bladder models. Segments, 1 cm in length, were cut directly below the catheter eyehole and attached to aluminum holders using carbon tape. The lumens of the catheters were imaged using a Hitachi TM-3030 Tabletop SEM operated at 5–15 kV.

### 2.5. Statistical analysis

Statistical analysis was performed using Graph Pad Prism 8.0 for Mac (GraphPad Software Inc., San Diego, USA). A Kruskal–Wallis test and post-hoc analysis using Dunn's multiple comparison test were performed to assess the effect of tEDTA on bacterial viability in AU and urinary pH after 6 and 24 h. Statistical differences in time to catheter blockage were determined using a one-way analysis of variance (ANOVA) and differences in pH and bacterial viability at the start of the bladder model experiment and at the control endpoint were evaluated using a two-way ANOVA. Post-hoc analysis using Tukey's multiple comparison test was performed. In all cases *n* ≥ 3 and *p* < 0.05 was used to denote significance.

## 3. Results and discussion

### 3.1. Analysis of tEDTA kill kinetics

*P. mirabilis*, *S. aureus*, and *E. coli* commonly colonise the urine and catheters of long-term catheterised patients.^25,26^ tEDTA minimum inhibitory and bactericidal concentrations against these uropathogens were determined (details provided in ESI† section S1.1). These assays were performed in broth media as per CLSI standards; however, tEDTA displays strong affinity to chelate divalent ions present in urine, with chelation of Ca^2+^ or Mg^2+^ in a 1 : 1 molar ratio, thus *in vitro* time kill kinetics of tEDTA were investigated in biologically-relevant AU.^7,15,19^ Chelation of Ca^2+^ and Mg^2+^ in AU consequentially reduces the concentration of tEDTA molecules available for interaction with bacteria; therefore, tEDTA concentrations of 7.63 mM (2.90 mg mL^−1^), representing the total concentration of Ca^2+^ and Mg^2+^ in AU, and 28 mM (10.65 mg mL^−1^), to provide excess tEDTA molecules to interact with the bacteria, were tested. At each time point, bacterial samples were neutralised with an excess of Ca^2+^ to ensure tEDTA inactivation through chelation.^20,21^ Preliminary studies confirmed the neutraliser neutralised tEDTA without affecting bacterial viability (details provided in ESI Section S1.2†).

**Fig. 3:**
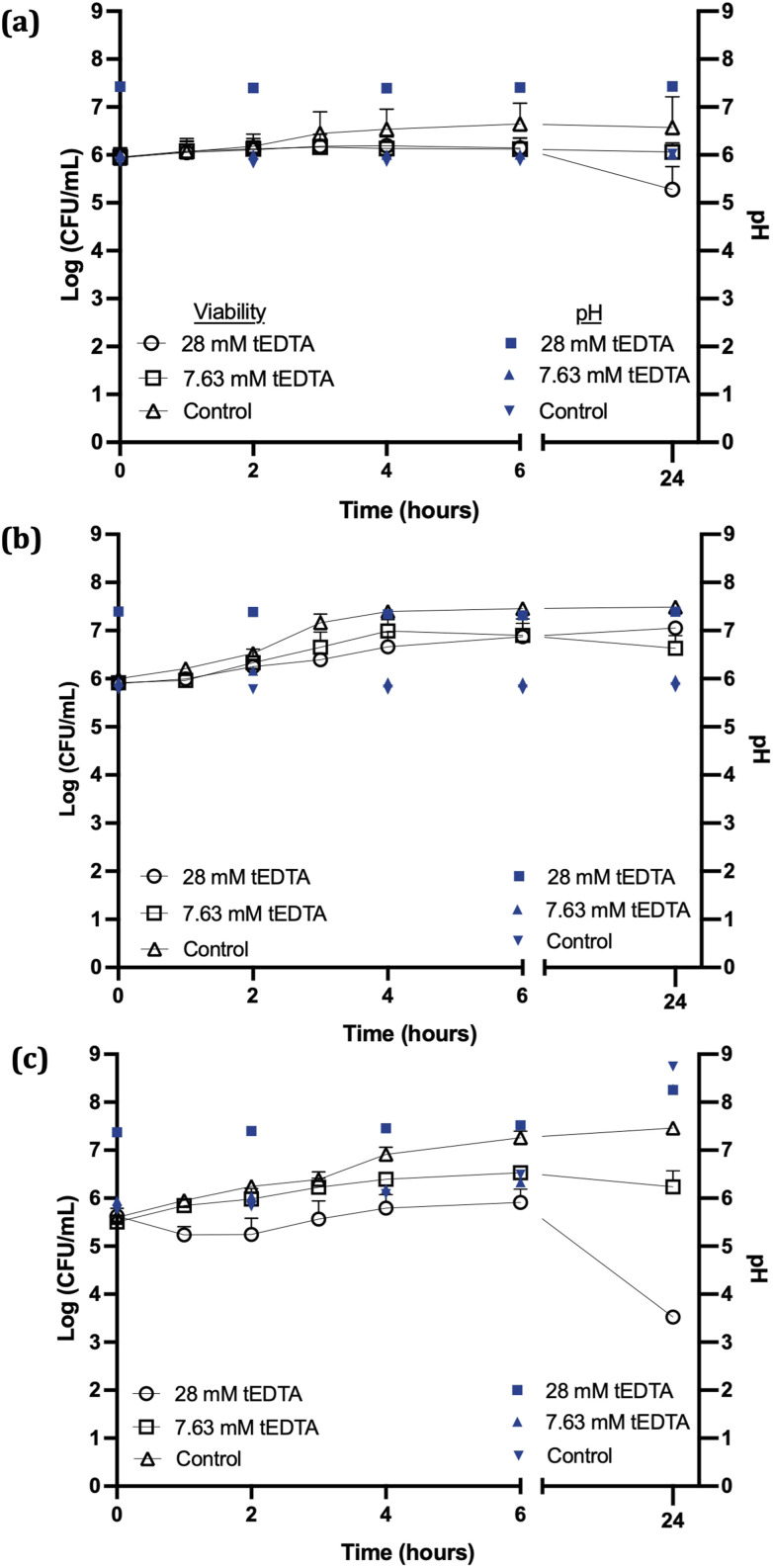
The pH (filled symbols) and viability (open symbols) of (a) *S. aureus*, (b) *E. coli* and (c) *P. mirabilis*-infected AU in the absence (control) and presence of tEDTA (7.63 or 28 mM). Error bars represent positive standard deviation (*N* = 3). Error bars not visible are less than the size of the symbols.

Fig. 3a shows no statistically significant reductions in *S. aureus* viability were observed following 24 h exposure to 7.63 or 28 mM tEDTA. Similarly, tEDTA had minimal antibacterial effect on *E. coli* (Fig. 3b). Conversely, Fig. 3c shows that *P. mirabilis* demonstrated greater susceptibility to tEDTA, with a 2.1 ± 0.2 log_10_ reduction in viability compared to the starting inoculum following 24 h exposure to 28 mM tEDTA. Statistically significant reductions of *P. mirabilis* compared to the control were observed in the presence of 28 mM tEDTA at 6 and 24 h.

EDTA's mechanism of action against Gram-negative and -positive bacteria differs.^9,11^ The outer membranes of Gram-negative cells contain divalent cations which stabilise the negatively-charged oligosaccharide chains of lipopolysaccharides (LPS) attached to the phospholipid bilayer.^9,27^ Chelation of these cations by EDTA destabilises the outer membrane causing release of LPS and subsequent solute leakage from the periplasmic space.^9,11^ In contrast, Gram-positive bacteria do not contain LPS or the associated divalent cations in their cell walls.^11,28^ While the mechanism of action of EDTA against Gram-positive bacteria remains poorly understood, studies suggest the target site for EDTA activity may be intracellular or EDTA potentially chelates ions required for biological processes essential for bacterial growth and survival.^12,13,21,29^

The lack of bactericidal activity observed with 7.63 mM tEDTA was due to the absence of sufficient free tEDTA molecules to interact with the bacteria and exert an antimicrobial effect. While the higher concentration of 28 mM tEDTA provided excess molecules for bacterial interaction, this was not sufficient for bactericidal activity against the challenge uropathogens, as defined by a ≥3 log_10_ reduction in bacterial density after 24 h exposure to tEDTA relative to the starting inoculum.^18,19^ However, the significant reduction in *P. mirabilis* viability in the presence of 28 mM tEDTA, relative to control suspensions, is promising as this is the pathogen primarily associated with catheter encrustation.^26^

**Fig. 4:**
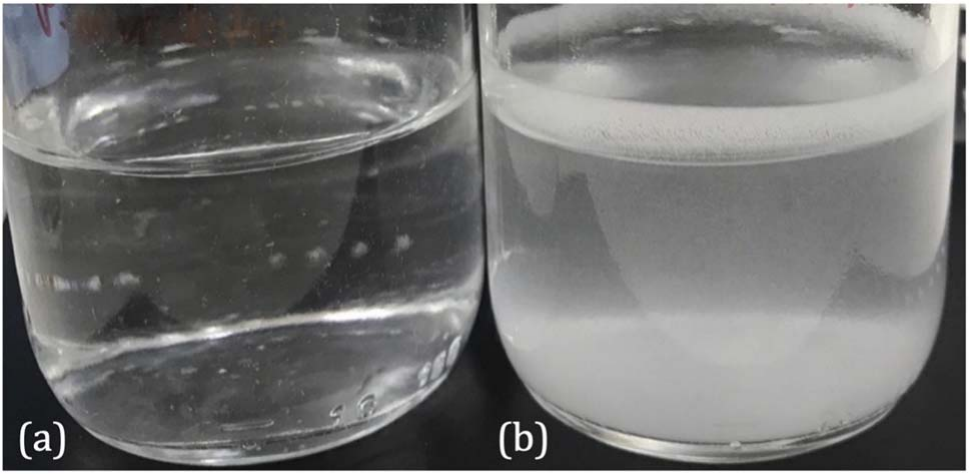
Images of *P. mirabilis*-inoculated AU after 24 h incubation at 37 °C in an orbital incubator in the presence of (a) 7.63 mM tEDTA and (b) 0 mM tEDTA.

The pH of the *P. mirabilis*-infected AU in the absence of tEDTA increased from pH 5.8 to 8.7 ± 0.2 by 24 h, because of the urease-catalysed hydrolysis of urea to ammonia and carbon dioxide. This caused precipitation of urinary salts (Fig. 4b) with carbonate apatite and struvite reported to precipitate at pH values exceeding 6.8 and 7.2, respectively.^7,15,30^ In contrast, as shown in Fig. 4a, *P. mirabilis*-infected AU solutions containing 7.63 and 28 mM tEDTA remained clear and precipitate-free despite the AU reaching pH values of 8.28, due to chelation of Ca^2+^ and Mg^2+^. Similarly, Prywer *et al.* reported prevention of both carbonate apatite and struvite formation due to chelation of all Ca^2+^ and Mg^2+^ in AU containing 10 mM disodium EDTA (dEDTA), whereas struvite formation was not completely inhibited with 5 mM dEDTA due to non-chelated Mg^2+^.^7^ This inhibition of crystallisation observed in the presence of both concentrations of tEDTA is promising with regards to use of this agent for combatting catheter encrustation due to crystalline biofilm formation.

It is important to note that, although a well-accepted AU medium was used in the *in vitro* studies,^17^ this medium does not account for the natural variation of urine composition between individuals. For example, excess iron can be excreted in the urine, which can be readily chelated by tEDTA, potentially reducing the concentration of tEDTA available to chelate Ca^2+^ and Mg^2+^, thus reducing its capacity to prevent carbonate apatite and struvite formation.

### 3.2. Struvite crystallisation studies

#### 3.2.1. Bulk crystallisation assays

Bulk crystallisation studies to assess the effect of tEDTA concentration on struvite nucleation and growth were performed in a simple growth solution. Calcium-containing components naturally present in urine were omitted to avoid precipitation of carbonate apatite. Rates of struvite crystallisation can be determined by monitoring the change of absorbance, pH, or conductivity of the growth solution over time.^7,31–33^ As shown in eqn (4), precipitation of struvite results in the release of protons, causing a decrease of solution pH.^31–33^

4 Mg^2+^ + NH_4_^+^ + H_2_PO_4_^−^ → MgNH_4_PO_4_·6H_2_O + 2H ^+^

The initial decrease in pH indicates the start of struvite nucleation and the rate of pH change is indicative of the rate of struvite formation.^31–33^ The corresponding decrease in pH during crystallisation allows the extent of reaction (EOR) to be monitored as a function of time.

**Fig. 5:**
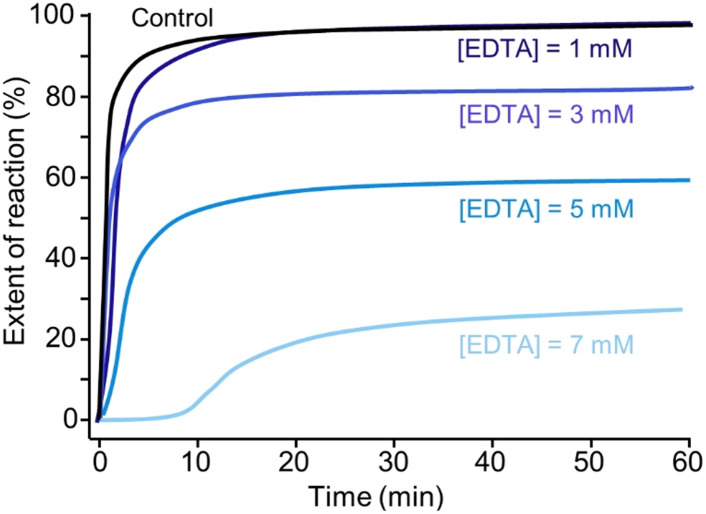
Extent of reaction of struvite formation in the absence (control) and presence of 1, 3, 5, and 7 mM tEDTA. These are representative curves from three trials for each sample, where characteristic features (*e.g.* induction times and extent of reactions) are reproducible.

Fig. 5a shows that crystallisation occurs almost instantaneously in growth solutions containing 0, 3, and 5 mM tEDTA. In the presence of 7 mM tEDTA there is an approximate 10 min delay before a gradual period of crystallisation, indicating a tEDTA concentration equivalent to that of the concentration of Mg^2+^ in solution (7 mM) is required to impede struvite nucleation. Similarly, dEDTA was found to impede struvite formation at near stoichiometric amounts compared to the Ca^2+^ and Mg^2+^ concentrations in solution.^7^ In contrast, potent inhibitors such as polyphosphates were shown to be effective at delaying or even completely suppressing struvite crystallisation processes at approximately two orders of magnitude less than the concentration of Mg^2+^.^34^ When the effects of an inhibitor on crystal growth are observed at low inhibitor concentrations, the common mode of action involves inhibitor adsorption on crystal surfaces, impeding incorporation of solute. Conversely, when inhibitor efficacy requires a concentration that is equivalent to or exceeds that of the solute concentration, the mode of inhibition is a thermodynamic effect; inhibitor-divalent ion complexation sequesters free Mg^2+^ in solution, thus lowering the supersaturation and reducing the rate of crystallisation.^8,35^

Furthermore, the presence of tEDTA influences the final pH of the solution. The reduction in the magnitude of total decrease in pH indicates the inhibitory effect of tEDTA on struvite crystallisation. With increasing tEDTA concentrations, the solution equilibrates at a higher pH corresponding to 0, 20, and 40% reductions in the EOR in the presence of 1, 3, and 5 mM tEDTA, respectively. In the presence of 7 mM tEDTA, the solution does not completely equilibrate over the extended screen time of 600 min but shows a gradual change in pH with >70% reduction in the EOR. To evaluate the thermodynamic contribution of the observed inhibition, equivalent measurements were performed at reduced magnesium concentrations in the absence of tEDTA to simulate Mg^2+^ sequestration by EDTA. These studies, which are detailed in ESI Section S2.3,† further indicate that the inhibitory effect on struvite nucleation and growth is achieved mainly through sequestration of Mg^2+^.

**Fig. 6:**
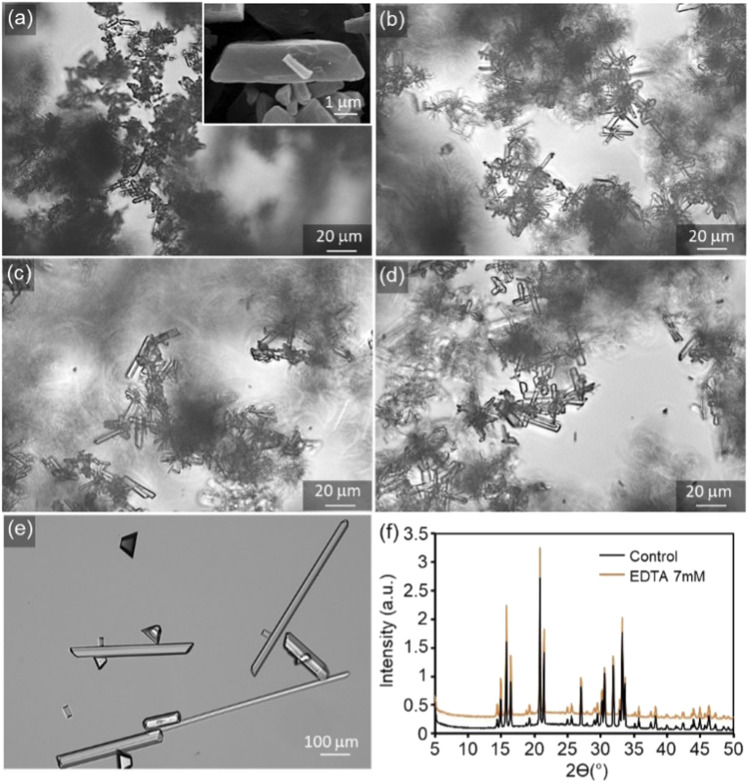
Representative optical micrographs of the struvite crystals formed during bulk crystallisation studies in the (a) absence (control) and presence of (b) 1 mM, (c) 3 mM, (d) 5 mM, and (e) 7 mM tEDTA (*N* = 6). The inset is a scanning electron micrograph highlighting the elongated tabular habit of a representative struvite crystal. (f) Powder XRD patterns for the control and 7 mM tEDTA samples.

Typically, net change in average crystal size, number density, and morphology of crystals obtained from bulk crystallisation assays serve as an indicator of the effects of crystal growth inhibitors. Without tEDTA, struvite crystals form an elongated tabular habit (Fig. 6a).^8^ tEDTA does not change the characteristic morphology of the crystal or the composition (Fig. 6f). However, there is a wide distribution of *b⃑*/*c⃑* and *b⃑*/*a⃑* aspect ratios in all samples which restricts the bulk crystallisation analysis. Furthermore, fewer, larger crystals formed in the presence of 7 mM tEDTA. The increase in size is typically attributed to two factors including growth promotion or reduction in nucleation density and the concomitant increase in size due to mass balance. In the presence of 7 mM tEDTA, it is most likely the latter case which is consistent with the significant reduction (>70%) in EOR observed in Fig. 5. Overall, it is difficult to distinguish between the effects on nucleation and crystal growth inhibition, and the heterogeneity in aspect ratio of crystals makes it difficult to quantify systematic trends in growth of struvite crystals as a function of tEDTA concentration.

#### 3.2.2. Microfluidics analysis of crystal growth inhibition

Using a microfluidics platform, the channels were seeded with struvite crystals to bypass nucleation and focus on quantifying growth rates under a constant flow of supersaturated solution. The growth solutions consisted of 2.5 mM NH_4_H_2_PO_4_ and 2.5 mM MgCl_2_·6H_2_O which was a metastable condition selected to avoid nucleation of new crystals and induce growth of crystal seeds in the microchannel.

**Fig. 7:**
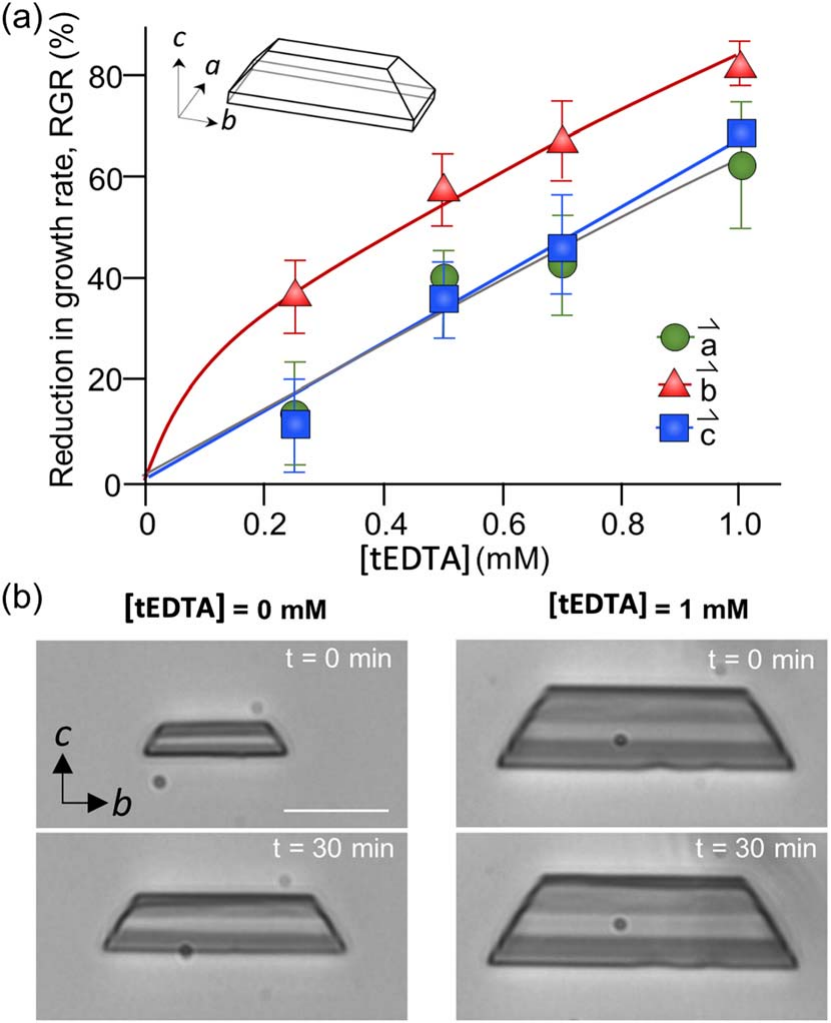
(a) Reduction in growth rate of struvite crystals along three crystallographic directions as a function of tEDTA concentration. The growth solution consisted of 2.5 mM MgCl_2_·H_2_O/2.5 mM NH_4_H_2_PO_4_/X mM tEDTA. (b) Time-elapsed optical micrographs demonstrated the effects of 1 mM tEDTA on struvite growth under solution flow (24 mL h^−1^). Scale bar = 20 μm. Each data point represents the average measurements of 30 or more crystals in a single batch using the microfluidics platform. Error bars span two standard deviations. The lines are interpolations to guide the eye.

The dependence of struvite growth on tEDTA concentration was analysed (Fig. 7a). The reduction in the relative growth rate (RGR) of struvite increased with tEDTA concentration in all three principal directions. This monotonic change in growth reduction with increasing tEDTA concentration indicates tEDTA does not bind to the crystal surface and instead impedes struvite growth through Mg^2+^ chelation. Moreover, the close to stochiometric amount of tEDTA required for a significant reduction in crystal growth rate confirms that the primary mode of inhibition is through ion sequestration from solution. The reduction in growth profile exhibits the following trend: *b⃑* > *c⃑* ≈ *a⃑*. The time-elapsed optical micrographs shown in Fig. 7b demonstrate struvite growth with no apparent change in crystal habit in the presence of 1 mM tEDTA, indicating tEDTA had no effect on struvite anisotropic growth under solution flow (24 mL h^−1^). A parallel experiment in the absence of tEDTA at a reduced magnesium concentration exhibted a similar profile (ESI Section 2.4†), further suggesting the growth inhibition is induced by decreasing Mg^2+^ rather than preferential binding of tEDTA to specific surfaces.

### 3.3. *In vitro* bladder model assays

Crystallisation studies indicate tEDTA concentrations equal to or exceeding those of the precipitating metal ions in solution can effectively impede struvite formation, suggesting the potential of tEDTA to be exploited in the prevention of urinary catheter encrustation. *In vitro* bladder model assays were performed with *P. mirabilis*-infected AU to investigate the effect of tEDTA on encrustation and blockage of indwelling urinary catheters.

**Fig. 8:**
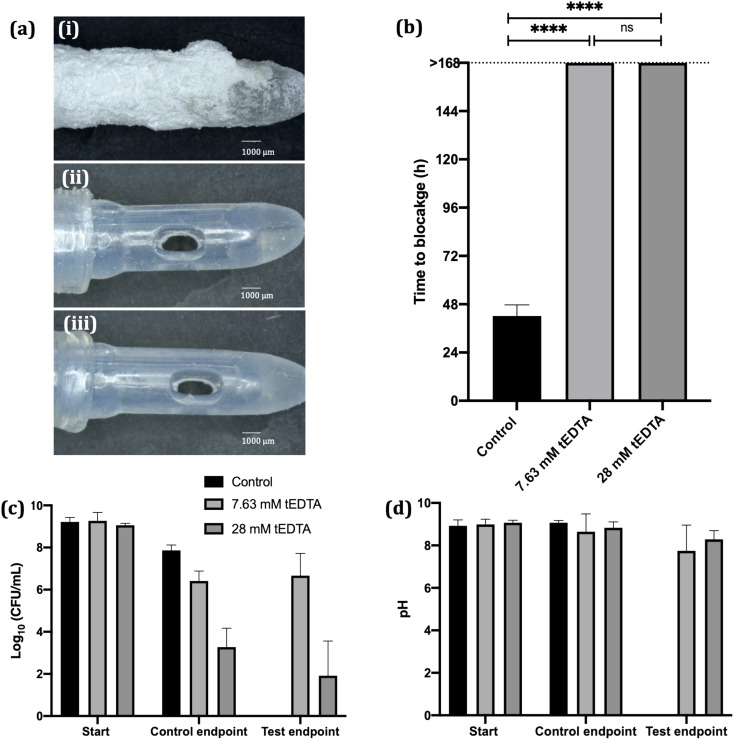
(a) Images of catheters supplied with *P. mirabilis*-infected AU in the (i) absence and presence of (ii) 7.63 mM and (iii) 28 mM tEDTA, removed from the bladder model at (i) time of blockage (42 ± 5 h) or (ii and iii) at 168 h. (b) Time taken for catheters to block when supplied with *P. mirabilis*-infected AU in the absence and presence of tEDTA. Catheters exposed to tEDTA remained unblocked throughout the duration of the experiment (168 h). **** denotes significance (*p* ≤ 0.0001) and ‘ns’ represents no significant difference. (c) *P. mirabilis* viability and (d) pH of urine within the bladders at the start of the experiment, time of catheter blockage and at *t* = 168 h. Error bars represent positive single standard deviations of the mean values (*N* = 6).

*In vitro* bladder model literature reports mean silicone catheter blockage times of 36–45 h when supplied with *P. mirabilis*-inoculated AU.^16,36,37^ Similarly, as shown in Fig. 8a (image (i)) and 8b, the control silicone Foley catheter supplied with *P. mirabilis*-infected urine, without tEDTA, blocked at 42 ± 5 h due to encrustation of the catheter and occlusion of the eyehole with a crystalline biofilm. In contrast, catheters exposed to tEDTA drained freely for 168 h, at which point the experiment was stopped, with no crystalline deposits present (Fig. 8a(ii) and (iii)). With a total of 7.63 mM Ca^2+^ and Mg^2+^ present in AU, tEDTA concentrations ≥7.63 mM were sufficient to chelate these polyvalent ions, preventing the formation of carbonate apatite and struvite crystals.

**Fig. 9:**
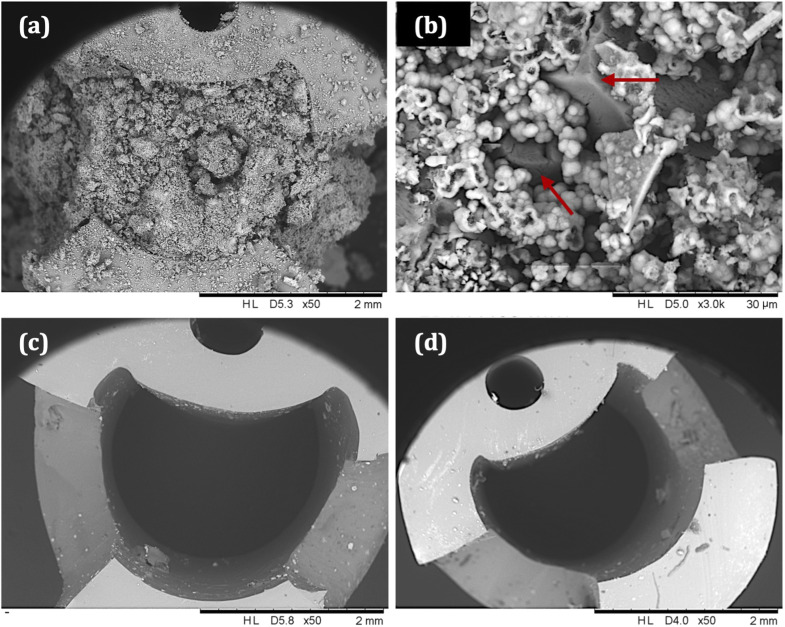
Representative scanning electron micrographs of catheter cross-sections directly below the catheter eyeholes. (a) A control catheter, supplied with AU without tEDTA, removed from the bladder model at the time of blockage (42 ± 5 h), with (b) displaying a magnified area of the deposits. Arrows have been added to highlight the struvite crystals present. Catheters supplied with AU containing (c) 7.63 mM tEDTA and (d) 28 mM tEDTA, removed from the bladder model at *t* = 168 h.

SEM images of catheter cross-sections (Fig. 9) further demonstrate the effect of tEDTA on catheter blockage. Fig. 9a shows the lumen of the encrusted control catheter at the time of blockage (42 ± 5 h) with the presence of crystalline deposits and 9b shows a magnified area of this material, displaying struvite crystals of faceted morphology surrounded by spherical carbonate apatite particles.^38^ As shown in Fig. 8d, the pH of residual urine within the control bladders exceeded pH 6.8 and 7.2, which is required for the formation of carbonate apatite and struvite crystals, respectively.^7^ Catheters exposed to 7.63 (Figure 9c) and 28 mM (Fig. 9d) tEDTA were free from encrustation at 168 h.

A concentrated (*ca.* 9 log_10_ CFU mL^−1^) *P. mirabilis* inoculum was used to inoculate the bladder models in order to emulate an established, high level of infection^36,39^*. P. mirabilis* viability within the control bladders maintained a high bacterial density (>7 log_10_ CFU mL^−1^). In the presence of 7.63 mM tEDTA, *P. mirabilis* viability remained around 6 log_10_ CFU mL^−1^ throughout the 168 h experiment. Conversely, 28 mM tEDTA had a bactericidal effect on *P. mirabilis*, significantly reducing bacterial density to 2–3 log_10_ CFU mL^−1^ within 48 h. Overall, tEDTA concentrations of ≥7.63 mM were capable of preventing encrustation and blockage of the catheters through chelation of Mg^2+^ and Ca^2+^ in AU, preventing the formation of carbonate apatite and struvite. Moreover, the higher concentration of tEDTA also had a bactericidal effect against *P. mirabilis* within the bladders, which may provide further benefit in preventing infection over longer periods, with indwelling catheters typically changed every 4–6 weeks.^40^

Using a similar bladder model set-up, Percival *et al.* reported daily catheter instillations of 80 mg mL^−1^ tEDTA decreased the rate of crystalline *P. mirabilis* biofilm formation and encrustation of Foley catheters, with mean blockage time delayed by *ca.* 22 h compared to the control (67 *versus* 45 h).^16^ This delay was attributed to dissolution of magnesium and calcium precipitates present in the catheter lumens during the 30 min daily instillation with tEDTA.^16^ Although our study employed a different approach, Fig. 9b confirms the absence of these precipitates within catheters continuously exposed to an approximately 27-fold lower concentration of tEDTA, with no evidence of catheter encrustation or blockage after 168 h. The fundamental research presented in this paper will inform our future development of a tEDTA-eluting urinary catheter coating, designed to allow the controlled, continual release of low tEDTA concentrations necessary to prevent the encrustation and blockage of indwelling catheters.

## 4. Conclusion

In this study we have shown that tEDTA exerts a bacteriostatic effect against *P. mirabilis*, a particularly problematic pathogen in the pathogenesis of catheter encrustation, as well as preventing precipitation of urinary salts. Struvite crystallisation assays confirmed the mechanism by which tEDTA impedes struvite growth is *via* chelation of Mg^2+^ in solution, with tEDTA concentrations equal to or greater than the Mg^2+^ required to prevent struvite formation. *In vitro* bladder model assays revealed a concentration of 7.63 mM tEDTA, equimolar to the concentration of divalent cations in urine, prevented encrustation and blockage of Foley catheters for over 168 h. With further research and optimisation, these promising initial findings suggest the clinical potential of this chelating agent, for example within an eluting catheter coating, to delay or prevent indwelling catheter blockage.

## Conflicts of interest

None declared.

## Supplementary Material

RA-013-D2RA06418A-s001
