# Supplementary material for: Tetrasodium EDTA for the prevention of urinary catheter infections and blockages
Source: RSC Adv. 2023 Jan 11;13(4):2202–12. doi: 10.1039/d2ra06418a (PMC9832581; doi:10.1039/d2ra06418a)
Supplement: RA-013-D2RA06418A-s001 [file RA-013-D2RA06418A-s001.pdf]

Electronic Supplementary Material (ESI) for RSC Advances.
This journal is © The Royal Society of Chemistry 2022

Supporting Information for:

Tetrasodium EDTA for the prevention of urinary catheter infections and blockages

Moore, J.V.[a], Kim, D.[b], Irwin, N.J.[a], Rimer, J.D.[b], McCoy, C.P.*[a]

[a]School of Pharmacy, Queen's University Belfast, 97 Lisburn Road, Belfast BT9 7BL, Northern Ireland, UK.

[b]Department of Chemical and Biomolecular Engineering, University of Houston, Houston, TX 77204, USA.

*To whom correspondence should be addressed. (e-mail: c.mccoy@qub.ac.uk)

Table of contents

Supporting methods
Supporting results
References

S1. Supporting methods

S1.1. Determination of minimum inhibitory and bactericidal concentrations of tEDTA

A broth microdilution method, based on the Clinical and Laboratory Standards Institute (CLSI) guidelines, was employed to determine the minimum inhibitory concentration (MIC) and minimum bactericidal concentration (MBC) of tEDTA against *S. aureus*, *P. mirabilis*, and *E. coli* [1,2]. Briefly, serial two-fold dilutions of tEDTA in MHB were performed in a microtiter plate. The inoculum was prepared by adjusting the turbidity of an overnight bacterial culture in logarithmic phase of growth to an optical density at 550 nm equivalent to 1 x 10^8 CFU/mL, as verified by viable count. The suspension was subsequently diluted to provide a final inoculum density of ca. 5 x 10^5 CFU/mL in each well. Following addition of the inoculum to each well of the plate, plates were incubated at 37°C in an orbital incubator at 100 rpm for 24 h. Positive and negative controls were included. The lowest tEDTA concentration that completely inhibited microbial growth was recorded as the MIC. Aliquots (20 µL) from wells exhibiting no visible growth were transferred onto nutrient agar (NA) or low swarm agar (LSWA) for *P. mirabilis*. The MBC was determined following 24 h incubation of the agar plates at 37°C as the lowest tEDTA concentration required to kill 99.9% of the inoculum. This assay was repeated on at least three independent occasions with triplicates of all concentrations and controls included on each occasion.

S1.2. Neutraliser toxicity and efficacy assessment

For the tEDTA kill kinetic and *in vitro* bladder model experiments, bacterial samples taken at each time point for analysis were serially diluted (ten-fold dilutions) in a neutraliser solution containing an excess of Ca^{2+} (3.20 mM) to ensure inactivation of residual tEDTA present, through chelation, thus preventing any further antimicrobial effect on the bacteria. Specifically, the neutraliser was composed of 2.66 mM calcium chloride dihydrate dissolved in QSRS (containing 0.54 mM Ca^{2+})[3–5]. Neutraliser toxicity and efficacy were evaluated to ensure the neutraliser solution effectively neutralised tEDTA without affecting the viability of the challenge microorganisms: *Staphylococcus aureus* ATCC 29213, *Escherichia coli* NSM59 and *Proteus mirabilis* ATCC 51286. The neutraliser toxicity assay involved preparing triplicate toxicity test and control solutions. The test solutions were composed of 0.5 mL bacterial inoculum ($1x10^8$ CFU/mL, suspended in artificial urine (AU)) in 4.5 mL of the neutraliser solution. The bacterial inoculum was prepared as detailed in §2.1. For the control solutions, phosphate buffered saline (PBS) was used in place of the neutraliser. Solutions were vortexed and incubated at room temperature for 10 min, and bacterial viability of the test and control solutions were determined using the Miles and Misra technique [6–8]. The neutraliser was considered nontoxic if there was $\leq 1 \log_{10}$ decrease in the number of viable cells in the test solution compared to the control solution [7,8].

The ability of the neutraliser to inactivate tEDTA activity was assessed by adding 0.5 mL 28 mM tEDTA solution (tEDTA dissolved in AU) to 4 mL neutraliser and vortexing. After 10 min, 0.5 mL of bacterial inoculum ($1x10^8$ CFU/mL) was added, the solution vortexed, left to incubate at room temperature for 10 min and then the bacterial viability determined. Triplicate efficacy control solutions were also prepared, with PBS in place of the neutraliser solution. The neutraliser was considered effective if there was $\leq 1 \log_{10}$ decrease in the number of viable cells in the neutralised test solution [7,8].

S1.3. Analysis of crystallisation kinetics

Equivalent kinetic measurements to those detailed in §2.2.2 were performed to assess the effect of varying magnesium concentration on the rate of struvite crystallisation in the absence of tEDTA. The 10 mL growth solution containing X mM $MgCl_2{\cdot}6H_2O$:7 mM $NH_4H_2PO_4$:150 mM NaCl (X=6.5, 6, 5, 4, 3, 2), adjusted to a final pH of 8.60 (±0.03) using 1 M NaOH, was prepared and stirred continuously. The pH was automatically recorded at 0.5 min intervals up to 600 min by an Orion 3-Star Plus pH benchtop meter equipped with a ROSS Ultra electrode (8102BNUWP). The

pH change of the growth solution was used as an indicator of struvite crystallisation (including the effects of both nucleation and crystal growth). The extent of reaction (EOR) was assessed according to the following equation:

$$EOR = \frac{(pH_i - pH_t)}{(pH_i - pH_{c,eq})} \times 100$$

(Equation 1)

Where pH_i is the initial pH (8.60), pH_t is the pH at time t, and $pH_{c,eq}$ is the final equilibrium pH (pH 7.42) achieved by struvite formation free of additive (control).

S1.4. Struvite microfluidics growth analysis

A microfluidics device was used to examine struvite growth following the set-up and protocol reported in previous work [9,10]. Equivalent measurements to those detailed in §2.3 were performed for various concentrations of magnesium in the absence of tEDTA. The growth solution composition was X mM $MgCl_2 \cdot 6H_2O$:2.5 mM $NH_4H_2PO_4$ (X = 2.5, 2.3, 2.0, and 1.5) with a pH of 8.60 (±0.03). The RGR was computed with respect to the control growth solution containing 2.5 mM $MgCl_2 \cdot 6H_2O$:2.5 mM $NH_4H_2PO_4$.

S2. Supporting results

S2.1. MIC and MBC determination

Minimum inhibitory and bactericidal tEDTA concentrations were determined against the Gram-positive *S. aureus,* and Gram-negative *P. mirabilis* and *E. coli*. The MIC and MBC values are presented in Table S1.

Table S1. MIC and MBC values of tEDTA against uropathogens.

	**tEDTA**	
	MIC mg/mL (mM)	MBC mg/mL (mM)
***P. mirabilis* ATCC 51286**	0.47 (1.24)	30 (78.9)
***E. coli* NSM59**	3.75 (9.86)	15 (39.5)
***S. aureus* ATCC 29213**	0.23 (0.60)	>80 (>211)

tEDTA displayed a bacteriostatic effect against *S. aureus* and *P. mirabilis* at lower respective concentrations of 0.23 and 0.47 mg/mL compared to *E. coli*, with a MIC of 3.75 mg/mL. The MIC values obtained for tEDTA against these microbes are in agreement with previous reports [11–16]. Furthermore, tEDTA exhibited bactericidal activity against the Gram-negative pathogens with MBC values of 15 and 30 mg/mL against *E. coli* and *P. mirabilis*, respectively. Conversely, tEDTA was not bactericidal against *S. aureus* up to the highest concentration tested (80 mg/mL). El-Sharif *et al.* reported the absence of biocidal activity of EDTA (salt not stated) against *S. aureus* and *E. coli* with concentrations up to 170 mg/mL [12].

S2.2. Neutraliser toxicity and efficacy assessment

Table S2. Control and test solution viable counts for the toxicity and efficacy assays

**Test solution**	***S. aureus*** **(log_{10} CFU/mL)**	***E. coli*** **(log_{10} CFU/mL)**	***P. mirabilis*** **(log_{10} CFU/mL)**
Toxicity control	7.37±0.03	7.31±0.05	7.63±0.01
Toxicity test	7.39±0.03	7.23±0.01	7.60±0.02
Efficacy control	7.40±0.02	7.27±0.03	7.57±0.03
Efficacy test	7.39±0.07	7.33±0.07	7.58±0.01

The Ca^{2+}-containing neutraliser had no toxic effects on the challenge microorganisms; there was ≤1 $\log_{10}$ decrease in the number of viable cells in the neutraliser test solution compared to the control solution, as shown in Table S2. Furthermore, the neutraliser effectively neutralised the antimicrobial action of the 28 mM tEDTA solution; there was ≤1 $\log_{10}$ decrease in the number of viable cells in the neutralised test solution [7,8].

S2.3. Analysis of crystallisation kinetics

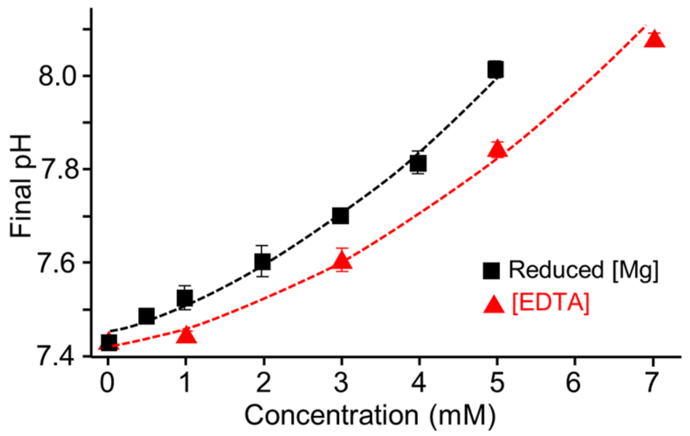

Figure S1. Final pH of the samples after complete crystallisation in the absence of tEDTA while varying the magnesium concentration (black square) and in the presence of varying concentrations of tEDTA ([Mg] fixed at 7 mM) (red triangle). Magnesium concentration is given as reduced [Mg] with respect to the magnesium concentration in the control (7 mM). Error bars not visible are less than the size of the symbols.

To evaluate the thermodynamic contribution of the observed inhibition, equivalent measurements to those discussed in §3.2.1 were performed at reduced magnesium concentrations in the absence of tEDTA to simulate Mg^{2+} sequestration by EDTA. As shown in Figure S1, as the magnesium concentration is reduced, the solution equilibrates at a higher pH, indicating a reduction in proton release, and therefore a reduction in crystallisation capacity. The final pH as a function of reduced magnesium concentration (Figure S1, black line) closely aligns with the final pH as a function of tEDTA concentration. This indicates that the inhibitory effect is achieved mainly through sequestration of Mg^{2+}. The slightly higher final pH induced by the reduction in magnesium concentration compared to the final pH at the equivalent concentration of tEDTA, indicates that the chelation ratio of Mg^{2+} to EDTA is less than 1 under these crystallisation conditions, which slightly differs from the known 1:1 chelation ratio [17,18].

S2.4. Struvite microfluidics growth analysis

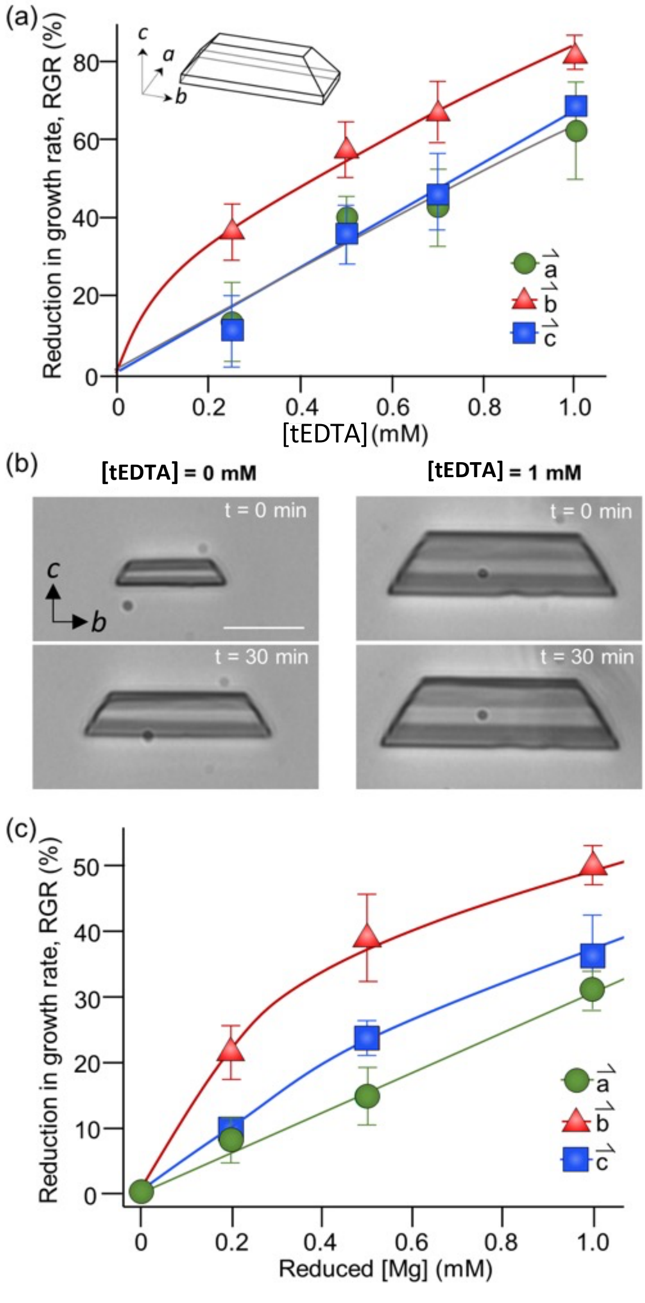

Figure S2 (same as Figure 7): (a) Reduction in growth rate of struvite crystals along three crystallographic directions as a function of tEDTA concentration. The growth solution consisted of 2.5 mM $MgCl_2 \cdot H_2O$/2.5 mM $NH_4H_2PO_4$/X mM tEDTA. (b) Time-elapsed optical micrographs demonstrated the effects of 1 mM tEDTA on struvite growth under solution flow (24 mL/h). Scale bar = 20 μm. (c) Reduction in growth rate as a function of magnesium concentration. Magnesium concentration is given as reduced [Mg] with respect to the magnesium concentration in control (2.5 mM). The growth solution consisted of X mM $MgCl_2 \cdot H_2O$/2.5 mM $NH_4H_2PO_4$. Each data point represents the average measurements of 30 or more crystals in a single batch using the

microfluidics platform. Error bars span two standard deviations. The lines are interpolations to guide the eye.

A parallel experiment to that detailed in §3.2.2 was performed in the absence of tEDTA at a reduced magnesium concentration to check if the characteristics of the profile were consistent (Figure S2c). In addition, the reduction profile exhibits the same trend of $\vec{b} > \vec{c} \approx \vec{a}$ with minimal difference in reduction for the $\vec{c}$ and $\vec{a}$ directions of growth. Therefore, the anisotropic reduction in growth rate in different directions of growth is most likely induced by decreasing Mg^{2+} rather than preferential binding of tEDTA to specific surfaces. Interestingly, there is an approximately 30% difference in RGR between the two profiles assuming a 1:1 Mg:tEDTA sequestration ratio. For instance, RGR in $\vec{a}$ with 1mM of tEDTA is ca. 80% compared to ca. 50% RGR when Mg is reduced by 1 mM. This indicates that, contrary to the bulk crystallisation assays, constant flow conditions facilitate magnesium sequestration and the sequestration ratio Mg:tEDTA is higher than 1.

References

[1] Clinical and Laboratory Standards Institute C. Methods for dilution antimicrobial susceptibility tests for bacteria That grow aerobically ; approved standard — ninth edition. Vol. 32. 2012.

[2] NCCLS. Methods for determining bactericidal activity of antimicrobial agents; approved guideline. CLSI document M26-A. NCCLS. 1999;19(18).

[3] Al-Bakri A, Othman G, Bustanji Y. The assessment of the antibacterial and antifungal activities of aspirin , EDTA and aspirin – EDTA combination and their effectiveness as antibiofilm agents. J Appl Microbiol. 2009;107:280–6.

[4] Arias-moliz MT, Ferrer-luque CM. Bactericidal activity of phosphoric acid , citric acid , and EDTA solutions against Enterococcus faecalis. Oral Surgery, Oral Med Oral Pathol Oral Radiol Endodontology. 2008;106(2):84–9.

[5] British Pharmacopoeia, Appendix XVI B. Microbiological examination of non-sterile Products [Internet]. [cited 2019 Apr 6]. Available from: https://www-pharmacopoeia-com.queens.ezp1.qub.ac.uk/bp-2019/appendices/appendix-16/appendix-xvi-b--microbiological-examination-of-non-sterile-products.html?date=2019-01-01

[6] Miles AA, Misra SS, Irwin JO. The estimation of the bactericidal power of the blood. J Hyg (Lond). 1938;38(6):732–49.

[7] Knapp L, Rushton L, Stapleton H, Sass A, Stewart S, Amezquita A, et al. The effect of cationic microbicide exposure against Burkholderia cepacia complex (Bcc); the use of

Burkholderia lata strain 383 as a model bacterium. J Appl Microbiol. 2013;115(5):1117–26.

[8] Irwin NJ, Bryant MG, McCoy CP, Trotter JL, Turner J. Multifunctional, low friction, antimicrobial approach for biomaterial surface enhancement. ACS Appl Bio Mater. 2020;3(3):1385–93.

[9] Kim D, Olympiou C, McCoy CP, Irwin NJ, Rimer JD. Time-resolved dynamics of struvite crystallization: insights from the macroscopic to molecular Scale. Chem - A Eur J. 2020;26(16):3555–63.

[10] Sosa RD, Geng X, Reynolds MA, Rimer JD, Conrad JC. A microfluidic approach for probing hydrodynamic effects in barite scale formation. Lab Chip. 2019;19(9):1534–44.

[11] Chan WY, Khazandi M, Hickey EE, Page SW, Trott DJ, Hill PB. In vitro antimicrobial activity of seven adjuvants against common pathogens associated with canine otitis externa. Vet Dermatol. 2019;30:133–8.

[12] El-Sharif AA, Hussain MHM. Chitosan – EDTA new combination is a promising candidate for treatment of bacterial and fungal infections. Curr Microbiol. 2011;62:739–45.

[13] Liu F, Hansra S, Crockford G, Köster W, Allan BJ, Blondeau JM, et al. Tetrasodium EDTA Is effective at eradicating biofilms formed by clinically relevant microorganisms from patients' central venous catheters. Am Soc Microbiol. 2018;3(6):1–16.

[14] Khazandi M, Pi H, Chan WY, Ogunniyi AD, Sim JXF, Venter H, et al. In vitro antimicrobial activity of ethylenediaminetetraacetic acid and polymyxin B nonapeptide against important human and veterinary pathogens. Front Microbiol. 2019;10:1–15.

[15] Sim JXF, Khazandi M, Pi H, Venter H, Trott DJ, Deo P. Antimicrobial effects of cinnamon essential oil and cinnamaldehyde combined with EDTA against canine otitis externa pathogens. J Appl Microbiol. 2019;127(1):99–108.

[16] Hamoud R, Zimmermann S, Reichling J, Wink M. Synergistic interactions in two-drug and three-drug combinations (thymol , EDTA and vancomycin) against multi drug resistant bacteria including E . coli. Phytomedicine. 2014;21(4):443–7.

[17] Prywer J, Olszynski M, Torzewska A, Mielniczek-brzóska E. Comparative in vitro studies on disodium EDTA effect with and without Proteus mirabilis on the crystallization of carbonate apatite and struvite. J Cryst Growth. 2014;395:123–31.

[18] Prywer J, Olszynski M. Influence of disodium EDTA on the nucleation and growth of struvite and carbonate apatite. J Cryst Growth. 2013;375:108–14.
